# Temporal Dynamics of Visually Induced Motion Perception and Neural Evidence of Alterations in the Motion Perception Process in an Immersive Virtual Reality Environment

**DOI:** 10.3389/fnins.2020.600839

**Published:** 2020-11-19

**Authors:** Min-Hee Ahn, Jeong Hye Park, Hanjae Jeon, Hyo-Jeong Lee, Hyung-Jong Kim, Sung Kwang Hong

**Affiliations:** ^1^Department of Otorhinolaryngology-Head and Neck Surgery, Hallym University College of Medicine, Anyang, South Korea; ^2^Laboratory of Brain & Cognitive Sciences for Convergence Medicine, Hallym University College of Medicine, Anyang, South Korea

**Keywords:** vestibular system, perception, motion sickness, sensory mismatch, virtual realtiy

## Abstract

Even though reciprocal inhibitory vestibular interactions following visual stimulation have been understood as sensory-reweighting mechanisms to stabilize motion perception; this hypothesis has not been thoroughly investigated with temporal dynamic measurements. Recently, virtual reality technology has been implemented in different medical domains. However, exposure in virtual reality environments can cause discomfort, including nausea or headache, due to visual-vestibular conflicts. We speculated that self-motion perception could be altered by accelerative visual motion stimulation in the virtual reality situation because of the absence of vestibular signals (visual-vestibular sensory conflict), which could result in the sickness. The current study investigated spatio-temporal profiles for motion perception using immersive virtual reality. We demonstrated alterations in neural dynamics under the sensory mismatch condition (accelerative visual motion stimulation) and in participants with high levels of sickness after driving simulation. Additionally, an event-related potentials study revealed that the high-sickness group presented with higher P3 amplitudes in sensory mismatch conditions, suggesting that it would be a substantial demand of cognitive resources for motion perception on sensory mismatch conditions.

## Introduction

Vestibular end organs initiate self-motion by sensing linear and angular accelerations rather than by sensing constant velocity (Lishman and Lee, 1973). However, humans can also perceive self-motion in a vehicle that is moving at a constant speed, which is generally mediated by the visual system (Mueller and Timney, 2016). Several studies have shown that visual signals processed in the primary visual cortex (V1) are relayed to higher-level visual processing regions, including the medial temporal gyrus (MT/V5), medial superior temporal region (MST+), dorsomedial area, cingulate sulcus visual area, precuneus motion area, parietoinsular vestibular cortex (PIVC), and ventral intraparietal region (VIP), which all contribute to motion perception (Duffy and Wurtz, 1991; Bradley et al., 1996; Brandt et al., 1998; Fasold et al., 2002). Moreover, cortical areas stimulated by visual signals are also activated by vestibular inputs (Brandt et al., 1998; Fasold et al., 2002), which suggests that dynamic sensory reweighting between these multisensory cortices could occur during motion perception. For example, a positron emission tomography (PET) study revealed significant deactivation of the visual cortex during caloric stimulation. In contrast, deactivation patterns in the vestibular cortices (PIVC and deep posterior insula) were observed during optic flow stimulation (Brandt et al., 1998).

Reciprocal inhibition of the visual and vestibular sensory systems operates to stabilize motion perception in the circumstances with a potential vestibular mismatch caused by incidental head rotation during locomotion in real-world situations (Brandt et al., 1998; Brandt and Dieterich, 1999). Therefore, the interactions of visual and vestibular inputs are a crucial element involved in the perception of motion in the real world, which involves a mixture of several types of motions (Hlavacka et al., 1992; Brandt et al., 1998; Gu et al., 2008; Fetsch et al., 2009).

Virtual reality technology has recently been implemented in many domains that extend beyond entertainment, including simulation programs, education, and various fields of medicine. However, in the virtual reality environment, many individuals suffer from cybersickness symptoms such as nausea, sweating, and dizziness that might result from mismatches between observed and expected sensory signals (i.e., the absence of vestibular signals). Even technical features, such as display characteristics, a field of view, and frame rate, partially influence the occurrence of cybersickness; sensory mismatches might be the most critical factor involved in the manifestation of this illness.

Therefore, the primary objectives of the present study were to explore spatio-temporal profiles of visually induced motion perception and to evaluate alterations in the motion perception process under a sensory mismatch condition using virtual reality. To accomplish this, an immersive virtual reality environment was used as an experimental platform to present sensory mismatch conditions and to simulate self-motion in the real world. Additionally, electroencephalogram (EEG) data were collected as a neurodynamic measure for temporal analyses.

## Materials and Methods

### Participants

EEG data were recorded from 20 healthy participants (five women) with a mean age of 29.7 years (age range: 21–48 years) in accordance with the ethics guidelines established by the Institutional Review Board of Hallym University College of Medicine and the Declaration of Helsinki (World Medical Association, 2014). All of the participants were right-handed, and none exhibited any abnormalities in conventional vestibular function tests. To normalize the cognitive abilities of the participants when performing the given task and to minimize distortion due to prior ear disease, participants were excluded if they (1) were older than 50 years of age, (2) had an active or prior history of vertigo and noise exposure, (3) were using ototoxic drugs, and/or (4) had a history of psychiatric problems.

After exposure to the immersive virtual reality environment, each participant was asked to complete the Korean version of the simulator sickness questionnaire (K-SSQ) that was translated from the original SSQ (Kennedy et al., 1993). Computation of the K-SSQ score was performed according to the scoring system suggested by Kennedy et al., wherein high susceptibility to cybersickness was defined as over 20 points in the total score (Kennedy et al., 1993). The data of three participants (one female) were excluded from the study due to excessive artifacts in the EEG data; therefore, 17 participants were included in the final analyses.

### Experimental Design

Two virtual driving conditions that simulated the view of a vehicle moving along a road (first-person perspective) were presented on the immersive virtual reality goggle system that was installed with an eye tracker (Oculus VR2 upgrade package; SensoMotoric Instruments, Teltow, Germany). The virtual driving simulations consisted of an acceleration condition (3.75 m/s^2^) and a constant speed condition (10 m/s) that were randomly presented for 6,000 ms. It was hypothesized that the accelerative visual motion would induce a sensory mismatch due to the absence of vestibular cues.

**FIGURE 1:**
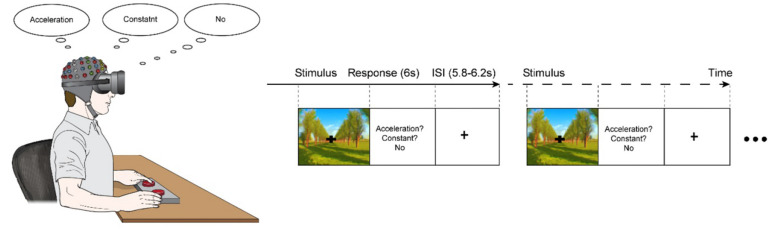
The experimental paradigm. A series of visual stimuli of either an acceleration of 3.75 m/s^2^ (acceleration-condition) or a constant speed of 10 m/s (constant-condition) were presented for 6,000 ms; the conditions appeared randomly with acceleration-condition of a 20% occurrence probability and constant-condition with an 80% occurrence probability. The experiment consisted of a velocity discrimination task, in which the participants were asked to press a button on one side if they perceived the speed to be changing (acceleration) and to press a button on the other side if they perceived the speed to be unchanging (constant). If the participants did not perceive any motion, they were instructed not to press any buttons.

Oddball task paradigm of 200 stimulus set was designed, which consisted of 40 accelerative driving simulation (20% occurrence probability in the stimulus set, target set) and 160 constant driving simulation (80% occurrence probability in the stimulus set, standard set), which were randomly presented. The interstimulus intervals (ISIs) randomly varied within a range of 5,800–6,200 ms, centered at 6,000 ms (Figure 1). During the ISI, the participants were instructed to discriminate between the virtual driving conditions by pressing a button on one side if an acceleration in speed was perceived (acceleration condition), pressing on a button on the other side if the speed was perceived to be unchanging (constant speed condition), and refraining from pressing either button if no motion was perceived. Trials that 1) the participant was fully fixating his/her vision on the fixation point displayed on virtual reality for consistency of behavioral response according to the sensitivity of motion sickness, and 2) provided a correct response were included for further analysis.

All contents of the virtual driving simulations were designed using Unity 3D (Unity Technologies, San Francisco, CA, United States) and Visual C# (Microsoft, Redmond, WA, United States) toolchains on a Windows 10 operating system. All test stimuli were displayed on a dedicated stimulus generation computer, and the collection of EEG signals was processed on a separate recording computer.

### EEG Acquisition and Signal Processing

A detailed description of the EEG acquisition and signal analysis procedures was described in a previous study by our research group (Hong et al., 2016). Briefly, all EEG data were acquired using a BrainAmp DC amplifier (Brain Products, Gilching, Germany) with an actiCAP consisting of 32 Ag/AgCl electrodes (Brain Products). The electrode placement was conducted in accordance with the International 10–10 system using the FCz as a reference and the AFz ground electrodes for recording. Electrode impedances were maintained below 5 kΩ before the recordings and the EEG data were recorded at 1,000 Hz. An analog band-pass filter was used to remove slow drifting artifacts, a low cut-off frequency of 0.1 Hz was used to remove muscular artifacts, and a high cut-off frequency of 70 Hz was used to remove line noise artifacts (frequency passing range: 0.1–70 Hz). Eye movement activity was monitored with an electrooculogram (EOG) electrode placed below the left eye, and vertical and horizontal electro-ocular activities were computed using two pairs of electrodes that were placed vertically and horizontally with respect to both eyes. More specifically, vertical activity was calculated as the difference between the Fp1 and EOG electrode signals and horizontal activity was calculated as the difference between the electrode signals from F7 and F8.

Bad channels were detected via visual inspection and then interpolated. EEG data were subsequently down-sampled to 512 Hz for connectivity and spectral power analysis, and 256 Hz for event-related potentials (ERPs) study, respectively. An independent component analysis was conducted to subtract artifactual components, including EOG data, cardiac signals, and muscular activity (Makeig et al., 1997). The EEG data were epoched into 5,700-ms periods, which included a pre-stimulus baseline of 1,000 ms. Bad epochs were automatically removed when they exceeded a transition threshold of 100 μV (Min-Max) where the maximally allowed gradient voltage step was 50 μV/ms.

### Estimation of Power Spectral Dynamics

To investigate the scalp distribution of neural stability, the EEG signals were spectrally decomposed using a standard fast Fourier transform algorithm over four-electrode clusters: frontal (F3, Fz, and F4), central (C3, Cz, and C4), parietal (P3, Pz, and P4), and occipital (O1, Oz, and O2). The mean powers for the responses (correct/incorrect) and conditions (acceleration/constant) were computed within a time window of 0–2,000 ms in the theta (4–7 Hz), low alpha (8–9 Hz), and high alpha (10–12 Hz) frequency bands, which are related to self-motion perception.

For the statistical analyses, the parameters from the included frequency bands (theta, low alpha, and high alpha), the four midline sites (frontal, central, parietal, and occipital areas from each spectral band), the driving simulation conditions (acceleration vs. constant), and the sickness level (high vs. low susceptibility) were analyzed with a repeated-measures ANOVA.

### Spatiotemporal Analysis Using the Granger Causality Method

A Granger causality analysis was performed to determine directional causal connectivity based on the electrophysiological signals elicited during the experiment (He et al., 2011); a previous study from our research group includes a more detailed background and description of this analysis method (Hong et al., 2016). In the present study, Granger causality was investigated at the grand-averaged evoked alpha activity. The Talairach coordinates of six bilateral regions of interest (ROIs) were manually selected: (1) the frontal eye field (FEF; right: *x* = 17, *y* = 23, *z* = 56; left: *x* = −18, *y* = 23, *z* = 56); (2) the PIVC (right: *x* = 50, *y* = −42, *z* = 18; left: *x* = −53, *y* = −42, *z* = 18); (3) the V1 (right: *x* = 10, *y* = −81, *z* = 6; left: *x* = −11, *y* = −78, *z* = 6); (4) the primary somatosensory cortex (S1; right: *x* = 39, *y* = −32, *z* = 55; left: *x* = −44, *y* = −31, *z* = 54); the (5) middle temporal area (MT; right: *x* = 29, *y* = −74, *z* = 7; left: *x* = −31, *y* = −73, *z* = 6); and (6) the VIP (right: *x* = 15, y−58, *z* = 59; left: *x* = −17, *y* = −58, *z* = 60). The ROIs were selected based on the standard brain area specifications from the Montreal Neurological Institute (Talairach and Tournoux, 1988) and cortical ROI functional connectivity was mapped with the directed transfer function (DTF) in the selected frequency components among the six ROIs using eConnectome software (He et al., 1987; He et al., 2011).

The arbitrary values of functional connectivity produced by the DTF function required assessments of statistical significance (He et al., 2011). Additionally, because the time series data and the DTF function derived from the data had highly non-linear relationships, the connectivity estimated by the non-parametric statistical test based on the surrogate data also required an evaluation of statistical significance (Ding et al., 2007). The surrogate data was Fourier transformed from the time series to an independent random shuffling of phases while conserving the magnitudes of the Fourier coefficients and then inversely transforming the magnitudes back into the time series. This phase shuffling process conserves the spectral characteristics of the time series and renders it suitable for DTF analysis to measure specific causal interactions in the frequencies. The process of shuffling the surrogate data and applying the connectivity estimates is repeated 1,000 times for each source of the time series dataset, which yields an empirical distribution of DTF values under the null hypothesis that implies the absence of causal connectivity (Ding et al., 2007). Accordingly, this statistical assessment of connectivity was performed in the present study using surrogate approaches (1,000 surrogate datasets, *p* < 0.05).

## Results

### Behavioral Data and Power Spectral Density

**FIGURE 2:**
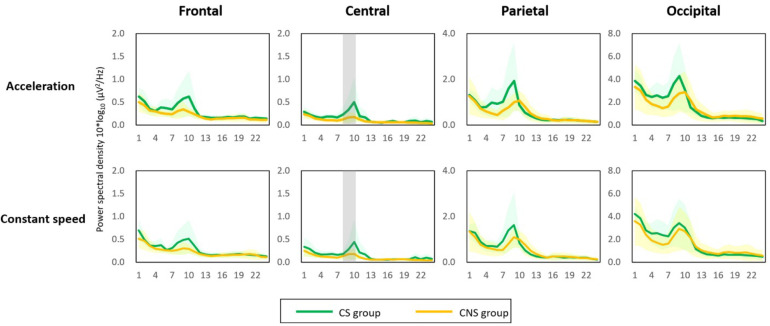
Power spectral density (PSD) results. An increase in low alpha PSD was observed in all brain regions and in both conditions (acceleration and constant speed). The low alpha PSD in the central area was significantly higher in the cybersickness susceptible (CS) group than in the cybersickness non-susceptible group for both conditions (*p* < 0.05). Gray shading = statistical significance.

The two types of visual stimuli presented on the virtual reality goggles consistently induced forward self-motion perception in all participants during the experiment. Of the 17 participants, 11 (64%) were highly susceptible to cybersickness based on a total score of K-SSQ and were assigned to the cybersickness-susceptible (CS) group. The power spectral density (PSD) in the low alpha frequency band relatively increased in all brain areas (frontal, central, parietal, and occipital clusters) and in both acceleration- and constant-speed simulation conditions (see Supplementary Table 1 for PSD results of all frequency bands and brain areas). In the acceleration condition, the low alpha PSD in the central area for the CS group (0.35 ± 0.29 μV^2^/Hz) was significantly higher than that in the cybersickness-non-susceptible (CNS) group (0.15 ± 0.08 μV^2^/Hz) (*p* < 0.05). Similarly, in the constant condition, the low alpha PSD was significantly higher in the CS group (0.30 ± 0.23 μV^2^/Hz) than that in the CNS group (0.16 ± 0.09 μV^2^/Hz; Figure 2) (*p* < 0.05). This finding indicates that neural activity in the alpha band might play an essential role in visually induced self-motion perception. Therefore, we explored spatio-temporal dynamics of alpha oscillations for visually induced self-motion perception.

### Brain Connectivity and ERPs

**FIGURE 3:**
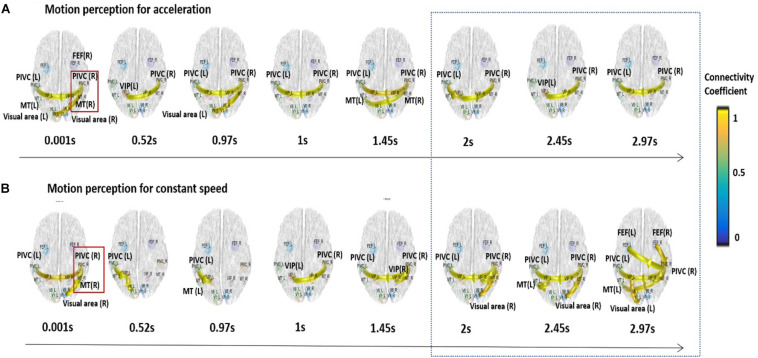
Dynamic functional connectivity for the temporal window of 3 s. Alpha band oscillations were initiated from the right PIVC following the presentation of the visual stimulation and then transmitted to the visual cortex regardless of visual stimulation velocity. More redundant connectivities were observed among the sensory cortices for the integration time window of 2–3 s in the constant condition **(A)**, while relatively scant connectivities were observed in the acceleration condition for the time window **(B)**.

**FIGURE 4:**
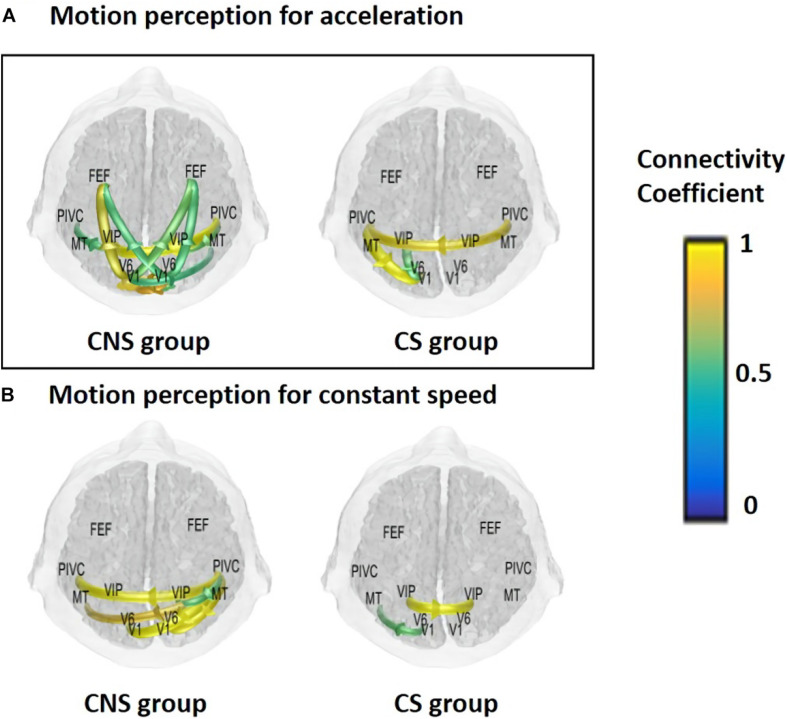
Mean functional connectivity associated with visual perception according to sickness susceptibility and the experimental conditions for a time window of 3 s. **(A)** Acceleration and **(B)** constant speed. CNS group had increased connectivities for the integration time window in both experimental conditions (CNS, cybersickness non-susceptible; CS, cybersickness susceptible).

Based on the previous analyses, alpha oscillations were used to investigate the neural dynamics of self-motion perception between the constant and acceleration conditions (sensory mismatch condition). The alpha band oscillations were instantly initiated from the right PIVC following visual stimulation and then transmitted to the visual cortex irrespective of the velocity features of the visual stimulus (acceleration vs. constant speed; Figure 3). However, there were some differences in neural dynamics for perception over time between the two conditions. In particular, we focused on neural dynamics for a time-window of 2–3 s because accumulated investigations suggested that the temporal integration window (TIW) of 2–3 s would be a crucial time for human cognition (Gibbon et al., 1997; Pöppel, 1997; Fairhall et al., 2014). Although a definite conclusion could not be reached, the results indicated that there were more redundant brain connectivities among the sensory cortices over time in the constant condition, whereas relatively scant connectivities were observed in the acceleration condition. We speculated that activation of different sensory cortices may contribute to stable motion perception. Interestingly, the mean temporal dynamics for the TIW also varied according to the cybersickness-susceptibility of the participants, in which the CNS group exhibited increased connectivity among motion sensory cortices as compared CS group in both visual stimuli conditions (constant and acceleration speed; Figure 4). These results suggest that alterations in dynamic connectivity, especially the scant connectivities among motion-sensitive cortices, were more present in the sensory conflict condition (acceleration condition) and CS group; therefore, these alterations were likely related to the cybersickness susceptibility.

**FIGURE 5:**
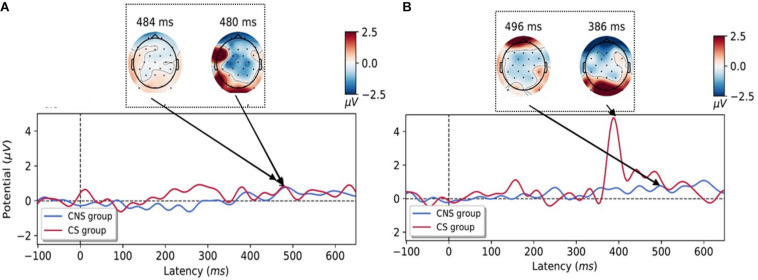
Grand-averaged event-related potentials (ERPs) time courses for the perception of constant-**(A)** and accelerative speed condition **(B)** on the occipital area. ERPs were depicted according to the cybersickness-non-susceptible (CNS) group (blue line) and cybersickness-susceptible (CS) group (red line) and the topographies during the processing of motion perception in an experimental task. Increased P3 amplitudes were observed in the CS group for motion perception of accelerative speed (CNS, cybersickness non-susceptible; CS, cybersickness susceptible).

ERPs to each condition were taken from −100 to 650 ms to capture the P3 component (Figure 5). For motion perception of constant speed, there are no significant differences of P3 amplitude (CS group: 0.89 ± 1.16 μV, CNS group: 2.08 ± 2.74 μV). However, CS group has significant higher P3 amplitudes (5.23 ± 5.48 μV) than those of CNS group (0.70 ± 1.05μV) for motion perception of acceleration speed condition (*p* < 0.027, t = −2.43), which seem to reflect more significant cognitive loading for processing of information for motion perception of accelerative condition (sensory conflict condition).

## Discussion

The present study was primarily concerned with exploring dynamic neural connectivity during the spatiotemporal integration of visually induced motion perception; more specifically, this study assessed the alterations in motion perception that are induced under sensory-conflicted situations in the absence of vestibular cues. The findings would provide novel insights into the neural mechanisms underlying cybersickness. This study employed a first-person driving simulation using immersive virtual reality technology with a vehicle that was moving either at a constant or accelerating velocity and was the first to assess neurodynamic measures with a high temporal resolution for motion perception. As motion perception is typically coupled with vestibular sensations in real-world situations, the visual stimulation with an accelerative component in the virtual reality environment should induce sensory conflicts due to the absence of vestibular signals.

The present analysis of the neural dynamics associated with motion perception using EEG data revealed that the alpha PSD in the central area in the CS group was significantly higher than that in the CNS group under the acceleration condition. This result showing a substantially higher PSD in the alpha band of the CS group is consistent with previous findings demonstrating that a higher degree of motion sickness is accompanied by increased activation in the alpha band (Chuang et al., 2016; Kim et al., 2019).

Spatiotemporal cortical behaviors induced by an optic flow can provide substantial physiological evidence regarding how the human brain interprets visual signals to help determine the relationship between internal representations and body position in space when dynamic motion stimuli are mixed up. Previous studies have reported that cortical areas that selectively respond to optic flow contribute to visual motion perception (Cardin and Smith, 2010) and that the PIVC is deactivated while experiencing motion perception induced by optic flow (Brandt et al., 1998; Brandt and Dieterich, 1999). However, these previous imaging studies were not designed to evaluate the temporal behavioral aspects involved in the processing of motion perception. That is, these earlier studies employed PET or functional magnetic resonance imaging (fMRI) techniques to explore the interaction between visual and vestibular signals by simply presenting visual stimuli as the radial or circular movement of a white dot on a black background. However, temporal changes in the brain during the induction of self-motion sensation may not have been fully captured due to the limited temporal resolution that those types of scanners provide. Additionally, since the event-related perception process is achieved due to activity within a complex neuronal network that is sequenced over time among different brain areas (Cohen, 2008; Schutter and Knyazev, 2012), the results of previous fMRI or PET studies may be limited in that they only reflect static functional connectivity based on inter-regional statistical analyses. Another problematic issue is that these previous investigations did not provide an immersive full-field environment for visually induced motion perception.

We focused on the exploration of spatiotemporal profiles, not those static neural connectivities for motion perception. Thus, an immersive virtual reality environment was used as the experimental platform for full-field visual stimulation, and that EEG data were used for the temporal analyses, which are the strengths of the present study.

We explored the neural dynamics of the alpha oscillations because this frequency band is the most prominent oscillation during visual motion perception in the current study. The results revealed that, following the presentation of visual stimulation, the alpha band oscillations were instantly initiated from the right PIVC and then transmitted to the visual cortex for both types of visual stimuli (acceleration and constant speed).

Since ongoing alpha oscillations involve a top-down inhibitory control process (Klimesch et al., 2007; Bazanova and Vernon, 2014), the PIVC seems to receive inhibitory signals following visual stimulation. This notion is consistent with the theory of reciprocal inhibitory visual-vestibular interactions that is typically understood as a functional sensory reweighting mechanism (Brandt et al., 1973, 1998; Dieterich et al., 1998). Nevertheless, variability between motion-perceptions of the two-speed conditions in terms of temporal dynamics was noted over time, in which there were increased connectivities among motion-sensitive cortices under the constant velocity condition for a crucial integrated time window (Gibbon et al., 1997; Pöppel, 1997; Fairhall et al., 2014). In contrast, there were fewer connectivities in the acceleration condition, which is our novel findings. Interestingly, scant dynamic-connectivities were consistently observed in the CS group regardless of the visual stimulus characteristics. We speculated that the different motor-sensitive cortices might operate cooperatively to reduce sickness, which may be presented as redundant connectivity in the CNS group. From those, we tentatively concluded that the temporal neural dynamics might be altered in the sensory conflict environment, contributing to cybersickness in sensitive individuals.

The ERPs analysis, which can provide high temporal resolutions in milliseconds, is an ideal tool to investigate alterations in the dynamic time course of neural connectivity for motion perception. Larger amplitudes of P3 are associated with superior information processing (Walhovd and Fjell, 2001; Polich, 2004) and the amplitude is proportional to the task complexity in healthy subjects (Gordeev, 2007). Our ERPs results revealed that the CS group had higher P3 amplitudes as compared to those of the CNS group in acceleration- condition (sensory mismatch condition, Figure 5).

If the brain may efficiently work for the cognitive process, it would not have the need to substantially recruit its cognitive capacity, resulting in P3 amplitude reduction. Our brain has an internal model updated with new information we experience in the world (Poon, 2004). Indeed, it is hard to perceive acceleration speed without vestibular sense in the real world. Thus, we speculated that our brain might need to substantially recruit cognitive resources for the perception in sensory conflict condition (acceleration-condition) because the condition might be new information to be updated in our internal model, which might affect the P300 amplitude in the CS group.

However, a primary concern still exists why neural alterations due to sensory conflict may not affect all subjects, which can be explained as an individual difference of internal model on the Basian brain (Poon, 2004; Egger et al., 2019). Presumably, when a certain level of motion perception for acceleration is reached, a more substantial proportion of cognitive resources is required for the updates of new information in CS group. In contrast, CNS group may have already updated the internal model for acceleration perception. Previous results confirmed that emotional stimuli enhance automatic processing, which results in larger amplitudes of ERPs (Carretié et al., 2004). Thus, we speculated a larger P3 amplitude could represent sickness level in sensory conflict situations.

However, these current results have some implicational limitations. First, source localization was conducted based on the limited number of scalp channels; thus, our estimates in dynamic connectivity should be understood with this possible problem of the EEG source localization. Second, our results should be restrictively interpreted with this statistical limitation into consideration due to the small sample size. Third, latency delay (−30 ms) and noise from the immersive virtual reality goggle system completely could not be corrected or removed due to methodological limitations. Thus, results from ERPs should be restrictively interpreted, but correction of the latency delay would not be a critical issue for the interpretation. Additionally, we did not investigate the adaptation process to long-term exposure to virtual reality.

Interestingly, changes in the mapping between perception and action in virtual reality (failure of perception-action coordination) have suggested a possible underlying pathology of cybersickness (Littman et al., 2010; Cook et al., 2018). This research found that behaviorally, people who are susceptible to motion sickness do not differentiate between motion situations, this seems to be similar to the ERP data of susceptible participants in the current study. As an alternative to visual-vestibular conflict theory, postural instability, in the presence of unexpected motion events (e.g., virtual reality situations), seems to be a substantial factor for the emergence of cybersickness.

## Conclusion

In conclusion, the present study showed that dominant inhibitory signals project out from the PIVC following visual stimulation, which is consistent with an earlier study (Brandt et al., 1998). Additionally, we found the neural alterations during visual motion perception in the sensory mismatch situation, which could be related to the generation of cybersickness. Since sensory conflicts have classically been accepted as the most prominent theory for explaining cybersickness, current technologies appear to be insufficient to provide a fundamental solution for preventing cybersickness in virtual reality without overcoming alterations in connectivities. Lastly, even we speculated that there are differences of sickness by the inherent internal model, the conclusion should be substantiated with further experiments.

## Data Availability Statement

The raw data supporting the conclusions of this article will be made available from the corresponding author, upon reasonable request.

## Ethics Statement

The studies involving human participants were reviewed and approved by the Institutional Review Board of Hallym University College of Medicine. The participants provided their written informed consent to participate in this study.

## Author Contributions

SKH: conceptualization. M-HA and JHP: data curation. M-HA and HJ: formal analysis. JHP: investigation, methodology, and resources. H-JK and SKH: supervision. H-JK, H-JL, and SKH: validation. SKH and JHP: writing – original draft. SKH and M-HA: writing – review and editing. All authors contributed to the article and approved the submitted version.

## Conflict of Interest

The authors declare that the research was conducted in the absence of any commercial or financial relationships that could be construed as a potential conflict of interest.
