# Supplementary material for: Temporal Dynamics of Visually Induced Motion Perception and Neural Evidence of Alterations in the Motion Perception Process in an Immersive Virtual Reality Environment
Source: Front Neurosci. 2020 Nov 19;14:600839. doi: 10.3389/fnins.2020.600839 (PMC7710904; doi:10.3389/fnins.2020.600839)
Supplement: Supplementary file 1 [file Table_1.docx]

**Supplemental Table 1. Differences in neural oscillatory power according to the general linear model results**

|  | **Theta** |  | **Low alpha** |  | **High alpha** | |  |  |
| --- | --- | --- | --- | --- | --- | --- | --- | --- |
| **FRONTAL** | | | | | |  |  |  |
| Group | F = 1.933, p = 185 |  | F = 2.738, p = .119 |  | F = 5.292, p = .036 * | |  |  |
| Condition | F = .002, p = .961 |  | F = 1.478, p = .243 |  | F = 1.167, p = .297 | |  |  |
| Group × Condition | F = .036, p = .853 |  | F = .001, p = .982 |  | F = .372, p = .551 | |  |  |
| **CENTRAL** | | | | | |  |  |  |
| Group | F = 3.322, p = .088 |  | F = 2.629, p = .126 |  | F = 3.974, p = .065 | |  |  |
| Condition | F = 2.446, p = .139 |  | F = 4.697, p = .047 * |  | F = 5.628, p = .031 * | |  |  |
| Group ⅹ Condition | F = 2.671, p = .123 |  | F = 11.110, p = .005 * |  | F = 4.208, p = .058 | |  |  |
| **PARIETAL** | | | | | |  |  |  |
| Group | F = 1.170, p = .296 |  | F = 2.259, p = .154 |  | F = .042, p = .841 | |  |  |
| Condition | F = 3.716, p = .073 |  | F = .447, p = .514 |  | F = 5.854, p = .029 * | |  |  |
| Group ⅹ Condition | F = .396, p = .539 |  | F = 5.398, p = .035 * |  | F = .278, p = .606 | |  |  |
| **OCCIPITAL** | | | | | |  |  |  |
| Group | F = 1.164, p = .298 |  | F = 1.877, p = .191 |  | F = .429, p = .522 | |  |  |
| Condition | F = 2.275, p = .152 |  | F = 5.347, p = .035 * |  | F = 7.208, p = .017 * | |  |  |
| Group ⅹ Condition | F = .193, p = .667 |  | F = 2.135, p = .165 |  | F = 1.547, p = .233 | |  |  |
